# Adverse events in families with hypertrophic or dilated cardiomyopathy and mutations in the *MYBPC3 *gene

**DOI:** 10.1186/1471-2350-9-95

**Published:** 2008-10-28

**Authors:** Philipp Ehlermann, Dieter Weichenhan, Jörg Zehelein, Henning Steen, Regina Pribe, Raphael Zeller, Stephanie Lehrke, Christian Zugck, Boris T Ivandic, Hugo A Katus

**Affiliations:** 1 Medizinische Klinik, Abt. Innere Medizin III, Universitätsklinikum Heidelberg, Germany; 2 Deutsches Krebsforschungszentrum, Heidelberg, Germany

## Abstract

**Background:**

Mutations in *MYBPC3 *encoding myosin binding protein C belong to the most frequent causes of hypertrophic cardiomyopathy (HCM) and may also lead to dilated cardiomyopathy (DCM). *MYBPC3 *mutations initially were considered to cause a benign form of HCM. The aim of this study was to examine the clinical outcome of patients and their relatives with 18 different *MYBPC3 *mutations.

**Methods:**

87 patients with HCM and 71 patients with DCM were screened for *MYBPC3 *mutations by denaturing gradient gel electrophoresis and sequencing. Close relatives of mutation carriers were genotyped for the respective mutation. Relatives with mutation were then evaluated by echocardiography and magnetic resonance imaging. A detailed family history regarding adverse clinical events was recorded.

**Results:**

In 16 HCM (18.4%) and two DCM (2.8%) index patients a mutation was detected. Seven mutations were novel. Mutation carriers exhibited no additional mutations in genes *MYH7*, *TNNT2*, *TNNI3*, *ACTC *and *TPM1*. Including relatives of twelve families, a total number of 42 mutation carriers was identified of which eleven (26.2%) had at least one adverse event. Considering the twelve families and six single patients with mutations, 45 individuals with cardiomyopathy and nine with borderline phenotype were identified. Among the 45 patients, 23 (51.1%) suffered from an adverse event. In eleven patients of seven families an unexplained sudden death was reported at the age between 13 and 67 years. Stroke or a transient ischemic attack occurred in six patients of five families. At least one adverse event occurred in eleven of twelve families.

**Conclusion:**

*MYBPC3 *mutations can be associated with cardiac events such as progressive heart failure, stroke and sudden death even at younger age. Therefore, patients with *MYBPC3 *mutations require thorough clinical risk assessment.

## Background

Hypertrophic cardiomyopathy (HCM) is a common disease with an estimated prevalence of 1:500 in north americans [1]. The disease may remain without clinical symptoms for many years. HCM has been recognized as the most common cause of sudden cardiac death in the young, especially in competitive athletes, where it accounts for 26% of cases [2]. Therefore, early diagnosis is crucial for prevention of such catastrophic events. For risk stratification, an expert based recommendation exists, which is mainly based on clinical findings such as maximum wall thickness [3], abnormal blood pressure response during exercise and non-sustained ventricular tachycardia [4]. Additionally, a positive family history for sudden cardiac death as a correlate of malignant gene mutations was recognized as an independent risk factor. Most cases of HCM are a result of mutations in genes encoding the sarcomeric proteins β-myosin heavy chain (*MYH7*), myosin binding protein C (*MYBPC3*), troponin T (*TNNT2*), troponin I (*TNNI3*), cardiac α-actin (*ACTC*) and α-tropomyosin (*TPM1*). Certain mutations in *MYH7 *and *TNNT2 *were ascribed a poor disease prognosis [5], while *MYBPC3 *mutations were attributed a more benign course and a low disease penetrance [6,7].

Dilated cardiomyopathy (DCM) is a major cause of heart failure in the young and accounts for about half of all heart transplantations. It is estimated that up to 35% of DCM cases are familial, indicative of potential gene mutations [8]. While HCM is mostly caused by mutations in sarcomeric protein genes, the genetic causes of familial DCM are more heterogeneous and hence, are less well defined. Up to 10% of familial DCM cases may be the result of mutations in sarcomeric protein genes like *MYH7*, *MYBPC3 *and *TNNT2 *[9]. Besides these genes, many others encoding cytoskeletal and nuclear envelope proteins, channel proteins and those encoding factors involved in calcium cycling were identified as candidates for DCM (for review [10]).

Here we studied the clinical outcome of families with HCM or DCM and mutations in *MYBPC3*, particularly with respect to adverse events like progressive heart failure, sudden cardiac death and stroke.

## Methods

### Patients

Patients presenting at the catheterization laboratory, cardiac MRI or outpatient department of our university hospital with a new or previously established diagnosis of a primary HCM or DCM were recruited for the study after written consent. Patients from external cardiologists were included, if a written consent and a blood sample was available. The diagnosis of primary HCM or DCM was established according to international standards. In short, HCM was defined by left ventricular wall thickening of 13 mm or more in the absence of secondary causes [11]. DCM was defined as depressed left ventricular ejection fraction less than 45% and a left ventricular end-diastolic diameter (LVEDD) of more than 117% of the expected value [12]. Echocardiography was performed in all patients according national and international standards. LVEDD was obtained from M-Mode recordings, maximum wall thickness and left ventricular ejection fraction (LVEF) form two dimensional echocardiography. LVEF was classified as follows: Normal ≥ 55%, mildly impaired 45–54%, moderately 30–44% and severely < 30% [13]. The study protocol was approved by the ethics committee of the medical faculty at the University of Heidelberg.

### Cardiac MRI

After exclusion of contraindications, cardiac MRI was performed in supine position at end-expiration employing an 1.5 Tesla, whole body superconducting MRI scanner system (ACHIEVA, Philips Medical Systems, Best, the Netherlands) using a five-element cardiac phased array surface coil with vector ECG-gating. Assessment of resting LV function was determined by cine images using a steady-state free precession sequence (matrix = 160/256, sense-factor = 2, flip-angle = 60°, slice thickness/gap = 8/2 mm) in continuous short axes covering the whole left and right ventricle from base to apex as well as 2-, 3- and 4-chamber views in anatomically correct heart axes. MRI data was transferred to a clinical workstation (Viewforum, Philips Medical Systems, Best, the Netherlands). End-diastolic (EDV, ml) and -systolic (ESV, ml) volumes and resulting ejection fraction (EF in %) were measured manually using short axes volumetry by determination of end-diastolic and -systolic cine images and subsequent delineation of endocardial borders excluding papillary muscles. For evaluation of myocardial mass, borders were also drawn at the interface between myo- and epicardium on end-diastolic images including papillary muscles. For anatomical description of late enhancement localisation, the AHA 17 segment model was employed consisting of six basal, six mid-ventricular, four apical segments as well as the apex. The signal intensities of late enhancement were described qualitatively (massive, normal, discrete) by consensus decision of the two observers.

### Mutation screening

87 patients with HCM and 71 patients with DCM were screened by denaturing gradient gel electrophoresis (DGGE) and subsequent sequencing for mutations in coding exons and flanking intron sequences of the *MYBPC3 *gene as described previously [14]. In an experiment of our previous study [14] testing the sensitivity of DGGE, 16 of 16 sequence verified variants among 187 different patient samples were accurately identified, indicating the excellent sensitivity of the method. All patients with mutations in *MYBPC3 *were additionally screened for mutations by single-strand conformational polymorphism analysis as already described [15] in cardiac troponin I (*TNNI3*; exon 8 screened by direct sequencing) or by DGGE in β-myosin heavy chain (*MYH7*; with the exception of exon 27), cardiac troponin T (*TNNT2*), cardiac α-actin (*ACTC*) and α-tropomyosin (*TPM1*; with the exception of exon 1). The primer sequences used in this study are available on request. To examine whether newly identified *MYBPC3 *mutations are present in "healthy" individuals and, therefore, to be considered as polymorphisms rather than as disease-causing mutations, 430 control persons with normal systolic and diastolic function were screened for these new mutations by DGGE. These controls were patients recruited from our cardiac catheterization laboratory, had no significant stenoses revealed by coronary angiography and no evidence for any myocardial disease

### Family screening

All first and second degree relatives of patients with a *MYBPC3 *mutation were asked to participate in our family screening program. Screening always included ecg and echocardiography. If an examination had been already performed by an external cardiologist, patients were asked for permission to request the examination record from their physicians. Furthermore, all relatives were invited to participate in the genetic screening and, if screened positive for a mutation, for additional cardiac MRI. This was performed in 16 of 42 mutation carriers. If possible, a pedigree of up to four generations together with a detailed family history was recorded. Deceased family members were considered affected if a sudden unexplained death occurred at or below 35 years of age [12]. Affection was presumed if a family member suffered from sudden death at 50 years of age or younger. Genetic counselling was offered to all participants and their families.

## Results

### MYBPC3 mutations in HCM and DCM patients

**Figure 1:**
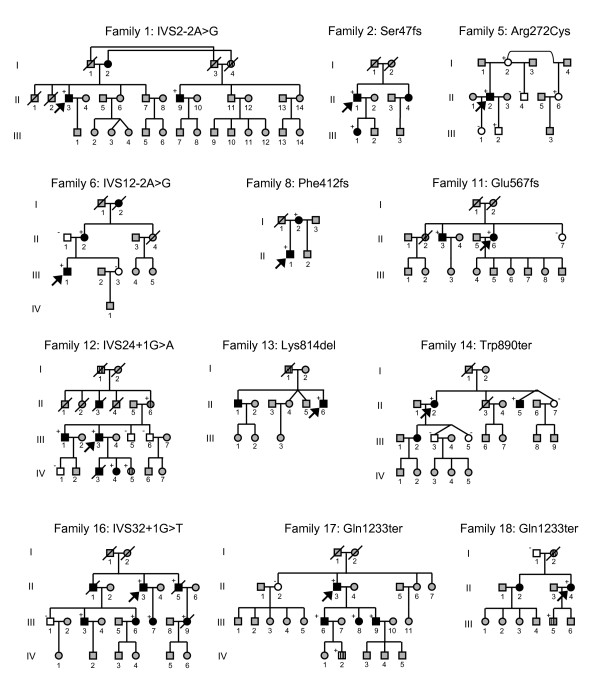
**Pedigrees of affected families: Open and black symbols represent unaffected and affected individuals, respectively.** Dashed symbols indicate individuals with presumed or borderline disease phenotype. Grey symbols denote individuals with unknown clinical status. (+) and (-) symbols indicate presence and absence of the mutation, respectively. Index patients are marked by an arrow. Slanted bars denote deceased individuals.

**Figure 2:**
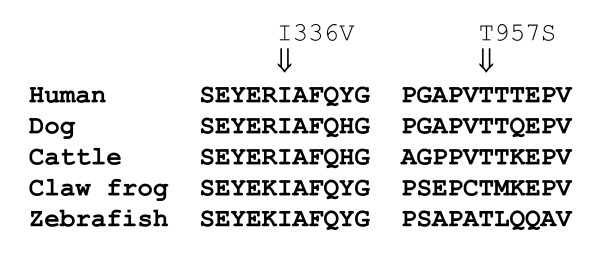
Conservation of mutated MYBPC3 amino acid residues throughout different species.

18 different mutations (four splice, three frameshift, seven missense, three nonsense mutations and one in-frame codon deletion), distributed throughout almost the entire gene, from intron 2 to exon 34, were found in 18 patients (Tables 1 and 2, Fig. 1). Close relatives of mutation carriers were subsequently genotyped for the respective mutation. Seven mutations were considered novel, since they were neither found in the large *MYBPC3 *mutation list of the "Cardiogenomics" database , nor in the SNP database dbSNP , nor in one of recent articles found in PubMed  describing novel mutations in the *MYBPC3 *gene. The causal role of the new mutations in the disease was underscored by their absence in 430 control individuals with normal systolic and diastolic function and, in one case (family 16), familial co-segregation with the disease. Moreover, four of the seven new mutations lead to a significant change of the amino acid sequence, namely two splice site mutations, one frameshift mutation and one nonsense mutation. A fifth mutation, p.R272C, altered one of three known phoshorylation motifs of the MYBP-C protein [16]. Preliminary analyses revealed a threefold decrease in the phosphorylation of a mutant p-R272C MYBP-C peptide fragment compared to the wild type fragment (unpublished data), yet the functional consequence of the mutation *in vivo *is still unknown. The two remaining missense mutations are considered functionally relevant since both affect highly conserved amino acid positions (Fig. 2).

**Table 1:** *MYBPC3 *mutations detected in this study

**No. of family or single patient**	Exon/IVS*	**Nucleotide position^† ^and change**	**Amino acid change**	**Affected**^‡^	**Genotyped total/pos.**	**Reference, comment**
1	IVS1	c.26-2A>G	unknown	3 (1)	2/2	Novel (similar [17])

2	2	c.141delT	p.S47fs	3	2/2	Novel

3	6	c.711C>A	p.Y237X	1	1/1	Novel

4	6	c.772G>A	p.E258K	1	1/1	[6,22,25-27]

5	7	c.814C>T	p.R272C	1 (1)	6/4	Novel^§^

6	IVS11	c.927-2A>G	unknown	2 (1)	4/2	[6,25]

7	12	c.1006A>G	p.I336V	1	1/1	Novel

8	15	c.1235_1236delTT	p.F412fs	2	2/2	[17,25]

9	17	c.1484G>A	p.R495Q	1	1/1	[6,17,28]

10	17	c.1505G>A	p.R502Q	1	1/1	[6,21,29]

11	18	c.1699_1670delGA	p.E567fs	3	3/2	[17]

12	IVS23	c.2308+1G>A	unknown	5 (3)	8/5	[19,25]

13	25	c.2437_2439delAAG	p.K814del	2 (1)	1/1	[17,27,29,30]

14	26	c.2670G>A	p.W890X	3	5/2	[17]

15	27	c.2870C>G	p.T957S	1	1/1	Novel

4	27	c.2873C>T	p.T958I	1	1/1	[31]

16	IVS31	c.3490+1G>T	unknown	7	7/6	Novel^§ ^(similar [32])

17	33	c.3697C>T	p.Q1233X	4 (1)	6/5	[18,19]; polymorphism [17]
					
18	33	c.3697C>T	p.Q1233X	3 (1)	3/2	

*IVS: intervening sequence; ^†^Numbering refers to the coding sequence of GenBank acc. no. U91629 starting with the A of start codon ATG as position 1; ^‡^additional relatives with suspected disease in brackets; ^§^DCM phenotype of the index patient

**Table 2:** Clinical characteristics of mutation carriers

No.*	**Indiv.**	**Sex**	**Age**	**Age at first presentation (Diagnosis)**	ECG***	**Echo**		**MRI**		
						
						***MWT*^† ^*(mm)***	***LVEF***^‡^	***MWT*^† ^*(mm)***	***LVEF (%)***	***Late enhancement***
1	II-3^§^	m	56	49 (HCM)	SR, CV	28	n	27	60	mid-, inferoseptal and inferior basal
	II-9	m	55	53 (HCM)	AF, LBBB	30	n-↓	33	51	massive septal, anterior, inferior
2	II-1^§^	m	55	55 (HCM)	pxAF	24	n			
	III-1	f	30	30^|| ^(HCM)	SR	15	n			
3	SP^#^	m	71	67 (HCM)	AF	20	n-↓			
4	SP^#^	m	48	46 (HCM)	SR, CV, iLBBB	25	↓			
5	I-2	f	85	n.a. (-)	AF	11	n			
	II-2^§^	m	65	62 (DCM)	SR, CV	15^††^	n	12.5	72^‡‡^	discrete septal (mid and basal)
	II-6	f	55	55^|| ^(-)	SR	10	n	< 11	65	none
	III-2	m	38	38 (susp.)	SR	14^††^	n	< 11	62^§*S*^	none
6	II-2	f	68	n.a. (HCM)	SR	n.a.				
	III-1^§^	m	39	18 (HCM)	SR	n.a.				
7	SP^#^	f	52	45 (HCM)	SR, CV	n.a.	n			
8	I-2	f	71	69^|| ^(HCM)	SR	20	n			
	II-1^§^	m	49	35 (HCM)	SR, AVB1, pxAF	25	↓	26	71	n.a.
9	SP^#^	f	66	44 (HCM)	SR, pxAF	24	n			
10	SP^#^	m	54	54 (HCM)	SR	22	n			
11	II-3	m	66	66^|| ^(HCM)	AF	20	n			
	II-6^§^	f	62	54 (HCM)	SR	22	n-↓			
12	II-6	m	67	67^|| ^(susp.)	SR, LAHB	12	n			
	III-1	m	46	42^|| ^(HCM)	SR	14	n	21	54	massive intramural basal- and mid-septal
	III-3^§^	m	42	37 (HCM)	SR	30	n			
	IV-4	f	20	20^|| ^(HCM)	SR	14	n	14	77	none
	IV-5	f	14	14^|| ^(susp.)	SR	13	n	11	66	none
13	II-1	m	54	54 (HCM)	SR	13	n			
	II-6^§^	m	42	21 (HCM)	SR	27	↓			
14	II-2^§^	f	61	57 (HCM)	SR	14^||^	n			
	II-5	m	55	55^|| ^(HCM)	SR	18	n	20	74	mid and basal septal
15	SP^#^	m	49	49 (HCM)	SR	19	n	24	62	anteroseptal basal
16	II-3^§^	m	66^##^	57 (DCM)	AF	n.a.^##^	↓↓↓^##^			
	II-5	m	65	34 (DCM)	AF, LBBB	12	↓↓↓			
	III-3	m	42	18 (HCM)	SR	15	n	22	67	intense septal
	III-6	f	40	40^|| ^(HCM)	SR	15	n			
	III-7	f	39	39^|| ^(HCM)	SR	15	n	14.5	72	septal, inferoseptal basal
	III-9	f	44	44^|| ^(HCM)	SR, iLBBB	16	n	22	71	midseptal and inferoseptal basal
17	II-3^§^	m	72	n.a. (HCM)	AF, RBBB	16	n			
	III-6	m	48	n.a. (HCM)	SR	24	n			
	III-8	f	43	43^|| ^(HCM)	SR	10	n	13.5	65	discrete basal-septal
	III-9	m	42	42^|| ^(HCM)	SR	18	n			
	IV-2	m	17	17^|| ^(susp.)	SR	12	n			
18	II-4^§^	f	37	27 (HCM)	SR	23	n	24	66	massive intramural basal- and mid-septal
	III-5	m	15	15^|| ^(susp.)	SR	12	n			

*Number of family or single patient (SP); ^†^MWT: maximum wall thickness; ^‡^Left ventricular ejection fraction (n: normal; ↓: mildly reduced, ↓↓↓: severely reduced); ^§^Index patient; ^||^First presentation due to screening program; ^#^single patient; **n.a.: not available; ^††^marked trabeculation; ^‡‡ ^End diastolic volume 205 ml, EDV index 94.9 ml/m^2 ^(reference value 47–92 ml/m^2^); ^§§ ^End diastolic volume 212 ml, EDV index 98.1 ml/m^2^; ^|| ||^4 years after transcoronary ablation of septal hypertrophy; ^##^heart transplantation with 58 years; ***ECG: electrocardiogram; SR: sinus rhythm; CV: status after electrical cardioversion due to atrial fibrillation; AF: atrial fibrillation; pxAF: paroxysmal AF; LBBB: left bundle branch block; iLBBB: incomplete LBBB; AVB1: first degree atrio-ventricular block; LAHB: left-anterior hemiblock; RBBB: right bundle branch block

Two new mutations were found in DCM patients of families 5 and 16, while all other mutations were detected in patients with HCM. One patient was a double-heterozygote for two different missense mutations located in exons 7 and 28. Members of the two unrelated families 17 and 18 had the same Gln1233ter nonsense mutation, which terminates the MYBP-C protein prematurely, leading to loss of 42 of the 1274 amino acids. This mutation had been described previously as a polymorphism [17], based on its presence in 2% of 200 phenotypically uncharacterized control individuals. We and others, however, considered the mutation disease-causing, since it was neither detected in 350 controls in previous studies [18,19], nor in our 430 controls. Moreover, the mutation clearly segregated with the disease in all five mutation carriers of family 17, either associated with a HCM phenotype or with borderline myocardial hypertrophy (IV-2).

To exclude mutations in other genes of high relevance in cardiomyopathies, the 18 initial mutation carriers were screened additionally for mutations in *MYH7*, *TNNT2*, *TNNI3*, *ACTC *and *TPM1 *encoding β-myosin heavy chain, cardiac troponin T, cardiac troponin I, cardiac α-actin and α-tropomyosin, respectively. No further mutations were found in these genes.

### Phenotypic heterogeneity of mutation carriers

We observed a broad spectrum of phenotypes among the mutation carriers: from asymptomatic and phenotypically normal at the age of 85, like I-2 of family 5 (Table 2), to heavily affected at a relatively young age, like III-9 of family 16 at the age of 44 or IV-3 of family 12 at the age of 13 (Tables 2 and 3). Even close relatives carrying the same mutation displayed a striking phenotypic heterogeneity, as documented by members of families 5, 12, 16 and 17.

**Table 3:** Adverse events in families of *MYBPC3 *mutation carriers

**Fam or single patient**	**Patient**	**Confirmed mutation carrier**	**Age at event**	**Type of event**
1	II-9	Yes	54	Transient ischemic attack

2	II-1	Yes	55	Stroke
	
	II-4	No*	49	ICD^†^

6	I-2	No*	35	Sudden death

7		Yes	51	Stroke

8	II-1	Yes	49	Pacemaker (Sick sinus syndrome)

9		Yes	59	Palpitations, positive EPS^‡^, ICD^†^

11	I-2	No*	55	Sudden death
	
	II-2	No*	36	Sudden death

12	I-1	No*	42	Sudden death
	
	II-3	No*	33	Sudden death
	
	II-6	Yes	67	Inferior MI, coronary heart disease
			67	ICD^†^: Non-sustained VT (holter)
	
	III-3	Yes	42	ICD^†^: Non-sustained VT (holter)
	
	IV-3	No*	13	Sudden death

13	I-1	No*	64	Stroke
			67	Sudden death
	
	II-1	No*	53	Transient ischemic attack

14	I-1	No*	53	Sudden death

16	II-1	No*	32	Sudden death
	
	II-3	Yes	58	Heart transplantation
	
	II-5	Yes	61	Resuscitation due to ventricular fibrillation, ICD^†^
			67	Heart failure related death
	
	III-9	Yes	45	Sudden death

17	II-3	Yes	64	Stroke

18	I-2	No*	42	Sudden death

*First degree relative with mutation; ^†^ICD: implantable cardioverter defibrillator; ^‡^EPS: electro-physiological study (programmed ventricular stimulation)

The index patient of family 5, II-2, initially presented with decompensated heart failure during new onset of tachyarrhythmic atrial fibrillation and arterial hypertension. In addition, cardiac catheterization ruled out significant coronary artery disease, but revealed an enlarged left ventricle with severely depressed systolic function. The patient improved, however, under guideline-conform treatment, as documented four years later by cardiac MRI displaying normalized left ventricular function and only minor pathological findings regarding wall thickness and myocardial late contrast enhancement. The son, III-2, had a mild enlargement of the left ventricle with an end-diastolic volume index of 98.1 ml/m^2 ^during MRI (reference value 47–92 ml/m^2^). However, he and his father showed a marked trabeculation on cardiac MRI. This finding did not fulfil the criteria for myocardial noncompaction, but might be an explanation for the overestimated wall thickness by echocardiography. Two further mutation carriers of family 5, I-2 and II-6, were asymptomatic and, as revealed by cardiac MRI, showed no phenotypical abnormalities.

In family 16, the index patient, II-3, and his brother, II-5, had a DCM phenotype. In contrast, the other mutation carriers, all belonging to the third generation of family 16, displayed a HCM phenotype upon cardiac MRI examination, yet were otherwise asymptomatic. Only in individual III-3, a HCM was diagnosed before during a routine physical examination.

The index patient of family 12 (III-3) had a severe left ventricular hypertrophy with outflow obstruction, which required septal ablation. His son (IV-3), who suffered from sudden cardiac death at the age of 13 years, also had a severe hypertrophy. In contrast, the index patient's mother (II-6), also mutation carrier, had only a borderline hypertrophy. In this patient, a comorbidity with a coronary heart disease and previous inferior infarction was prevalent, and the ventricular arrhythmias found at holter monitoring might be a result from a myocardial scar after inferior infarction.

In family 17, the index patient, II-3, presented at the age of 72 years with a leading finding of a restrictive pathophysiology and only a mild wall thickening, which probably was a result of an end-stage HCM. Unfortunately, high-quality cardiac MRI recording was impossible due to atrial fibrillation. All three children of this patient were mutation carriers; only one was previously diagnosed with HCM, while the other son and the daughter were diagnosed with HCM in the course of our family screening.

### Adverse clinical events

Malignant clinical events such as unexplained sudden death, stroke or transient ischemic attack were ascertained in many members of our study families (Table 3). Based on twelve families and six single patients, a total number of 42 mutation carriers was identified, of which eleven had at least one adverse event. A total number of any adverse event occured in 23 of 45 (51.1%) cardiomyopathy cases. Eleven individuals of seven families suffered from sudden death between 13 and 67 years of age. In three families, a sudden death occurred at or before the age of 35 years. Any adverse event occurred in eleven of twelve families and in two of six single mutation carriers. The youngest, IV-3 of family 12, already diagnosed with an extreme septal hypertrophy and a dynamic outflow obstruction, died at the age of 13. Moreover, a first degree relative, II-3, of a mutation carrier in the same family experienced sudden death at the age of 33. In family 16, a sudden death occurred only shortly after the diagnosis of HCM had been ascertained in the course of family screening. The female patient, III-9, had no heart failure symptoms but a maximum wall thickness of 22 mm, moderate late enhancement during contrast MRI and mild elevated NT-proBNP. Clinical findings in the patient did not qualify for an implantable cardioverter defibrillator (ICD) based on available risk scores [4]. A formerly asymptomatic individual, II-1, of the same family suffered from sudden cardiac death at the age of 32 in relation to exercise. This patient had not been genotyped, yet can be considered mutation carrier based on the fact that his two brothers, II-3 and II-5, and two symptomatic children, III-3 and III-6, are mutation carriers.

Five patients of four families received an ICD for primary prevention because of non-sustained ventricular tachycardia or other high-risk disease profiles. Stroke or a transient ischemic attack related to atrial fibrillation occurred in six patients of five families between 51 and 64 years of age. Two of these patients had persisting disabling symptoms. Two patients with stroke and two with a transient ischemic attack recovered without permanent impairment.

### Genotype-phenotype correlation

Sudden death as the most serious adverse event was prevalent in seven of 18 families (39%). However, despite missense mutations were most frequent with seven of 18 index cases, none of the sudden deaths was observed in families of these patients. In contrast, sudden deaths occurred in three of four families with splice mutations, in two of three with nonsense mutations, in one of three with frameshift mutations and in the single family with an in-frame codon deletion. All three families with sudden deaths at or before the age of 35 years were carriers of splice mutations.

## Discussion

Mutations in the *MYBPC3 *gene are among the most prevalent causes of inherited HCM. In earlier studies, e.g. [6,7], *MYBPC3 *mutations were found to be associated with a low penetrance, late onset and a benign course of the disease. This notion became challenged, however, by the findings of two recent studies. In the first, carriers of a specific *MYBPC3 *frameshift mutation from 15 unrelated families revealed a high penetrance, early onset and malignant course of the disease [20]. In the second, a high proportion of *MYBPC3 *mutations was found in children with HCM [21]. Here we screened 87 patients with HCM and 71 patients with DCM for mutations in *MYBPC3 *and correlated the genotypes, phenotypes and clinical outcomes of the mutation carriers and their close relatives. We identified 18 different mutations in 18 index patients, 16 with HCM and 2 with DCM. Mutations in other likely candidate genes for HCM and DCM were excluded. In addition to the 18 index patients, 24 more *MYBPC3 *mutation carriers were detected among relatives, distributed over ten families. In ten of them, the presence of a cardiomyopathy was unknown before. 16 of the 24 additional mutation carriers displayed a clear disease phenotype. Additional three individuals had a borderline phenotype: One of them had hypertension as potential cause of secondary hypertrophy and coronary artery disease. Two more and still young individuals, a 17 year old male (IV-2 of family 17) and a 15 year old female (III-5 of family 18), showed also borderline signs of the disease. Only three mutation carriers, all members of family 5, had no certain pathological findings. The corresponding mutation, a novel p.R272C missense mutation, altered one of three known phoshorylation motifs of the MYBP-C protein [16]. We found decreased phosphorylation at this position *in vitro*, but we don't know the functional consequences *in vivo*. In this family, only the index patient showed a DCM phenotype, for which a different cause, e.g. hypertensive heart disease, cannot be ruled out. The other family with a DCM phenotype of the index patient was family 16 with a novel splice site mutation. Two older mutation carriers suffered from DCM, while four younger patients from the following generation either showed symptoms of HCM or at least a HCM phenotype upon cardiac MRI examination. It is most likely that the two older patients suffered from an end-stage HCM with progression to a DCM phenotype, as had been described already in a few cases of *MYH7 *and *MYBPC3 *mutation carriers [22]. For still unknown reasons, the number of *MYBPC3 *mutations associated with DCM is much lower [23] than the number of *MYBPC3 *mutations associated with HCM.

Contrasting to previous findings in families with *MYBPC3 *mutations [7], malignant clinical events were frequently observed in our families, even at a young age. Sudden cardiac deaths at or even before the age of 35 did occur in four members of families 6, 12 and 16. In accordance with current recommendations [4], these data underscore the importance of a close meshed risk stratification in every patient at risk due to a mutation in *MYBPC3*. Although the number of mutations is too small to prove statistically significant, it is remarkable that all sudden deaths at or before the age of 35 occurred in families with splice mutations, while no sudden death was observed in families with missense mutations. Stroke and peripheral embolizations had been reported by others in 6% of HCM patients, with an incidence of 0.8% per year [24]. As several factors in HCM are favouring manifestation of atrial fibrillation, it is not surprising that in five of 18 families a history of stroke or transient ischemic attack was prevalent. Therefore, clinical care of patients and family members at risk should consider risk indicators for atrial fibrillation like left atrial enlargement, severe diastolic dysfunction or family history of stroke or high susceptibility for atrial fibrillation.

A major limitation of our study is that genotype and phenotype data of deceased family members are not available in most cases. At a more advanced age, alternative causes of sudden death like myocardial infarction and atrial fibrillation become more likely. Therefore, only a well documented sudden death below 35 years of age is an accepted criterion for familial involvement in a cardiomyopathy [12]. In our retrospective evaluation of affected families, it cannot be excluded, that other conditions may have led to the adverse events. Nevertheless, it is noteworthy that in many of our families adverse events are prevalent in first-degree relatives at both older and younger ages, while *MYBPC3 *mutations initially were believed to be associated with a good prognosis. However, a referral bias in our observational study cannot be excluded, although the reason for presentation in virtually all index patients were diagnostic procedures to rule out concomitant coronary artery disease, but not adverse events. Mutations in other likely candidate genes for HCM were not found in any of the index patients. As to the heterogeneity of the genetic causes of cardiomyopathies, additional mutations in less likely or hitherto unknown disease genes cannot be excluded. Moreover, yet unknown disease modifiers might have had a modulating effect on the disease phenotype. The question of the prognostic impact of certain mutations in cardiomyopathies has to be clarified by inclusion of mutations carriers into registries.

## Conclusion

The finding of a *MYBPC3 *gene mutation is not generally associated with a good prognosis. Therefore, *MYBPC3 *mutation carriers should be as well subjected to careful clinical risk stratification.

## Competing interests

The authors declare that they have no competing interests.

## Authors' contributions

PE and DW conceived and designed the study. JZ and RZ carried out mutation detection. HS and SL were responsible for the cardiac imaging data. RP and CZ acquired and interpreted the clinical data. DW, BTI and HAK handled funding, supervised the study and manuscript drafting. The manuscript was written and approved by all authors.

## Pre-publication history

The pre-publication history for this paper can be accessed here:


